# Histopathological and Molecular Predictors of the First Site of Dissemination in Non-Small Cell Lung Cancer

**DOI:** 10.3390/curroncol32110617

**Published:** 2025-11-04

**Authors:** Vlad-Norin Vornicu, Alina-Gabriela Negru, Razvan Constantin Vonica, Andrei Alexandru Cosma, Daniela-Sonia Nagy, Mihaela Maria Pasca-Fenesan, Anca Maria Cimpean

**Affiliations:** 1 Doctoral School in Medicine, “Victor Babes” University of Medicine and Pharmacy, 300041 Timisoara, Romania; vlad.vornicu@umft.ro (V.-N.V.); cosma.andrei@umft.ro (A.A.C.); mihaela.fenesan@umft.ro (M.M.P.-F.); 2 Department of Oncology-OncoHelp Hospital Timisoara, Ciprian Porumbescu Street, No. 59, 300239 Timisoara, Romania; 3 Department of Cardiology, “Victor Babes” University of Medicine and Pharmacy, 300041 Timisoara, Romania; alinanegru@umft.ro; 4 Preclinical Department, Faculty of Medicine, “Lucian Blaga” University of Sibiu, 550169 Sibiu, Romania; razvanconstantin.vonica@ulbsibiu.ro; 5 Department of Microscopic Morphology/Histology, “Victor Babes” University of Medicine and Pharmacy, 300041 Timisoara, Romania; acimpeanu@umft.ro; 6 Department of Oncology, Faculty of Medicine, “Victor Babes” University of Medicine and Pharmacy, 300041 Timisoara, Romania; 7 Center of Expertise for Rare Vascular Disease in Children, Emergency Hospital for Children Louis Turcanu, 300041 Timisoara, Romania; 8 Center of Genomic Medicine, “Victor Babes” University of Medicine and Pharmacy, 300041 Timisoara, Romania; 9 Research Center for Pharmaco-Toxicological Evaluation, “Victor Babes” University of Medicine and Pharmacy, 300041 Timisoara, Romania

**Keywords:** non-small-cell lung cancer, metastasis, histology, adenocarcinoma, squamous cell carcinoma, large-cell carcinoma, EGFR, KRAS, ALK

## Abstract

Background: Non-small-cell lung cancer (NSCLC) is often diagnosed at stage IV, when prognosis depends on metastatic spread. The impact of histopathology on the first metastatic site remains underexplored. Methods: We retrospectively analyzed 364 patients with stage IV NSCLC diagnosed at OncoHelp Medical Center, Timișoara, Romania (2020–2024). All underwent baseline CT chest–abdomen–pelvis, whole-body FDG PET-CT, and brain MRI within seven days of histological confirmation. Patients were stratified into adenocarcinoma (*n* = 164), squamous cell carcinoma (*n* = 112), and large-cell carcinoma (*n* = 88). The first metastatic site was defined as the earliest confirmed location. Associations were evaluated using Fisher’s exact test and multinomial logistic regression. Results: Histology was associated with the first metastatic site (global *p* = 0.013). Adenocarcinoma was more likely than squamous carcinoma to present with brain metastases (RRR 3.74, 95% CI 1.48–9.45; *p* = 0.005; *p*_FDR_ = 0.053) and showed directional signals toward bone and adrenal involvement. Squamous carcinoma more frequently spread to the pleura as the first site (adjusted *p* = 0.008). Large-cell carcinoma showed no consistent differences compared to squamous carcinoma. In adenocarcinoma subgroups, EGFR-mutant tumors most often metastasized to the brain (55.6%), KRAS-mutant tumors to the liver (44.4%), and ALK-rearranged tumors to bone (100%). Conclusions: The first metastatic site in NSCLC follows histology-specific patterns, with adenocarcinoma favoring hematogenous spread and squamous carcinoma showing locoregional involvement. Molecular status further refines these patterns in adenocarcinoma. Incorporating histology into baseline staging may improve diagnostic efficiency and prognostic accuracy.

## Simple Summary

Lung cancer is often discovered after it has already spread, and the location of the first metastasis can strongly influence treatment decisions. In this study, we analyzed patients with advanced non-small-cell lung cancer to see if the tumor’s histological type could predict the first site of spread. We found that adenocarcinoma was more likely to involve the brain, bones, or adrenal glands, while squamous cell carcinoma more often affected the pleura. Certain genetic changes in adenocarcinoma also showed specific patterns. These results may help doctors select the most useful imaging tests at diagnosis and personalize patient care.

## 1. Introduction

Lung cancer remains one of the most pressing health challenges worldwide, accounting for more deaths each year than breast, colon, and prostate cancers combined [1]. Despite major advances in early detection, molecular profiling, and targeted therapies, overall survival remains disappointingly low [2]. One of the main reasons for this is that most patients are diagnosed at advanced stages, when metastatic disease has already occurred [3]. For clinicians, anticipating where the first metastasis will appear is of relevance, as it not only reflects tumor biology but also determines the trajectory of disease progression, influences prognosis, and shapes surveillance strategies [4,5].

Among the many factors that drive metastatic spread, histopathology plays a central role [6]. Lung cancers are traditionally classified into two major groups: small-cell lung cancer (SCLC) and non-small-cell lung cancer (NSCLC). The latter, which represents about 85% of cases, includes adenocarcinoma, squamous cell carcinoma, and large-cell carcinoma, each with distinct morphological and clinical characteristics [7]. Adenocarcinoma, currently the most common subtype, typically arises in the lung periphery and tends to metastasize early to distant organs such as the brain, liver, and bones [8]. By contrast, squamous cell carcinoma usually originates centrally, showing a greater tendency for local and regional invasion before distant dissemination [9,10]. Large-cell carcinoma, although less frequent, is often aggressive and associated with variable metastatic patterns [11].

Importantly, metastasis is not a random event but follows preferential routes shaped by tumor biology, the tumor microenvironment, and genetic alterations [12,13]. For example, EGFR mutations have been associated with a predilection for brain involvement in adenocarcinoma [14,15], while KRAS mutations may favor hepatic spread [16]. Understanding the first site of metastasis is particularly critical, as it often dictates the initial clinical presentation, guides imaging and staging protocols, and may serve as an early indicator of prognosis [10,17].

Despite growing insights into the molecular and biological underpinnings of NSCLC, there remains a gap in translating histological differences into reliable predictions of where metastatic disease will first manifest. This knowledge is especially valuable in clinical settings where comprehensive molecular profiling is not available, and histopathology remains the cornerstone of decision-making.

Therefore, the aim of this study is to investigate the correlation between histopathological subtypes of NSCLC and their first metastatic site, with the goal of identifying clinically meaningful patterns that may refine prognostic assessment and optimize patient management.

## 2. Materials and Methods

### 2.1. Study Design and Setting

This investigation was conceived as a retrospective, single-center, observational study, carried out at the OncoHelp Medical Center in Timișoara, Romania, a regional referral oncology unit providing comprehensive cancer care. The primary aim was to evaluate the correlation between histopathological subtype of NSCLC and the first site of metastatic spread at diagnosis.

The study covered a 60-month interval, from 1 June 2022 to 31 April 2025, allowing for the inclusion of a well-defined cohort whose diagnostic and laboratory data were complete at baseline. The retrospective design reflects everyday oncology practice, making the findings directly applicable to real-world patient care.

### 2.2. Study Population and Subgroups

We conducted a retrospective, observational, single-center study at OncoHelp Medical Center, Timisoara, Romania. Patients were enrolled between 1 June 2022 and 31 April 2025. Reporting adheres to STROBE guidelines [18]. Tumor staging was assigned according to the TNM classification (8th edition), which was in effect during the study period [19]. Patients (n = 364) were stratified into three histopathological subgroups:

• Adenocarcinoma Group (N = 164)—ADCG—histologically confirmed pulmonary adenocarcinoma [20].
• Squamous Cell Carcinoma Group (N = 112)—SCCG—histologically confirmed squamous cell carcinoma [21].
• Large-Cell Carcinoma Group (N = 88)—LCCG—histologically confirmed large-cell carcinoma [22].

### 2.3. Inclusion and Exclusion Criteria

Adults (≥18 years) with histologically confirmed NSCLC (adenocarcinoma, squamous cell carcinoma, or large-cell carcinoma) and stage IV disease at diagnosis, in whom all baseline staging imaging was completed at diagnosis as follows:

• Contrast-enhanced chest–abdomen–pelvis CT.
• Whole-body FDG PET-CT.
• Brain MRI including, at minimum, T1, T2, FLAIR, DWI ± post-contrast T1 sequences, in line with current guidelines [23].

All three studies were performed within a ±7-day window of the date of histologic diagnosis and prior to initiation of any systemic anticancer therapy or radiotherapy. Patients in whom any required study was missing or nonconforming were excluded.

Were excluded from the study patients with synchronous malignancies that could confound dissemination patterns; hematologic malignancies; active systemic infections at the time of laboratory sampling; chronic autoimmune/inflammatory diseases or preexisting chronic immunosuppression prior to baseline assessment; prior systemic anticancer therapy before the initial workup; severe bone marrow suppression not attributable to lung cancer; incomplete records precluding determination of the first site; major contraindications to MRI or PET-CT that could not be overcome.

If two sites were unequivocally present, the site with the earliest documentation timestamp within the diagnostic window was chosen.

### 2.4. Data Collection

All relevant information was extracted retrospectively from the institutional electronic medical record system using a standardized protocol designed to minimize selection bias and ensure consistency across cases. The dataset was deliberately comprehensive, capturing both patient-related and tumor-related features, as well as laboratory and imaging findings at the time of diagnosis.

From a demographic and clinical perspective, the variables collected included age at diagnosis, sex, and place of residence (urban vs. rural), reflecting the diverse catchment population of the oncology center. Lifestyle factors such as smoking history, alcohol consumption, and occupational exposure to known carcinogens were recorded, together with comorbidities that could influence both prognosis and treatment options. The Eastern Cooperative Oncology Group (ECOG) performance status was systematically documented to provide an objective measure of baseline functional capacity [24].

Regarding tumor-related variables, data collection focused on histopathological subtype (adenocarcinoma, squamous cell carcinoma, or large-cell carcinoma) and the anatomical location of the primary tumor, categorized as central or peripheral. Disease staging was determined according to the TNM classification system in use during the study period [19]. Molecular profiling results were available, and they were incorporated into the dataset, particularly for key genetic alterations such as EGFR mutations [25], KRAS mutations [26], and ALK rearrangements [27], given their known implications for both prognosis and metastatic behavior.

During the early study period (2020–2022), molecular testing was performed selectively, with KRAS analysis mainly restricted to EGFR- and ALK-negative cases; from 2023 onward, reflex next-generation sequencing (NGS) became routine. Consequently, cases that were not tested for all three biomarkers (EGFR, KRAS, and ALK) were reclassified as unknown biomarker status rather than “wild type”. The designation “wild type” was reserved strictly for tumors that were tested and confirmed negative for all three biomarkers within the study’s molecular panel.

The pattern of metastatic dissemination was carefully recorded, with special attention to the first site of metastasis identified at diagnosis. This was confirmed by imaging methods (CT, MRI, PET-CT) and, in selected cases, by biopsy or histopathological examination. The analysis concentrated on common metastatic sites such as the brain, bone, liver, adrenal glands, pleura and contralateral lung. For consistency, only the first site of metastasis was considered in the main analysis, as this was central to the study objective.

All records were reviewed manually for accuracy, and data were anonymized prior to statistical analysis to ensure both scientific reliability and respect for patient confidentiality. The first metastatic site was defined by the organ with the earliest evidence of metastatic involvement at the time of diagnosis.

### 2.5. Ethical Considerations

The study complied with the Declaration of Helsinki and institutional research policies. The protocol was approved by the Ethics Committee of OncoHelp Medical Center, Timișoara (approval no. 1186/7 May 2025). As the research was retrospective and data were anonymized, informed consent was waived. All data were stored in secure, password-protected databases accessible only to the study team.

### 2.6. Statistical Analysis

Analyses were performed in GraphPad Prism 9 and IBM SPSS v27 (two-sided α = 0.05). Continuous variables were compared across histology using one-way ANOVA (or Kruskal–Wallis when assumptions were violated); categorical variables by Pearson’s χ^2^ or Fisher’s exact test. When a global test indicated differences, pairwise contrasts were adjusted for multiplicity using the Holm method within each variable.

The distribution of first metastatic sites across histologywas tested with the Fisher–Freeman–Halton exact test (3 × k). Pairwise 2 × 2 comparisons (ADC vs. SCC, SCC vs. LCC, ADC vs. LCC) are reported as raw and Holm-adjusted *p*-values within each site; inference is based on adjusted values.

The association between histology and first metastatic site was additionally assessed by multinomial logistic regression (reference: SCC; reference outcome: contralateral/intrapulmonary), with effects as relative risk ratios (RRRs) and 95% CIs. False discovery rate for the 10 component tests (5 sites × 2 contrasts) was controlled by Benjamini–Hochberg (reported as *p*_FDR_). Overall association was evaluated by the likelihood-ratio χ^2^ (G-test).

## 3. Results

Baseline comparisons across the three histopathological subgroups are presented in Table 1. While most demographic and clinical variables showed broadly similar distributions, a few meaningful differences emerged. Age varied slightly but significantly between groups, with post hoc analysis indicating that patients with adenocarcinoma tended to be younger than those with squamous carcinoma. Smoking status was the most striking differentiator: active smoking was far more prevalent among squamous cell carcinoma patients, with significant contrasts not only against adenocarcinoma but also large-cell carcinoma. By contrast, other parameters, including body mass index, performance status, and common comorbidities, did not differ substantially between groups, underscoring a largely comparable baseline clinical profile apart from smoking behavior and subtle age-related patterns.

Table 2 summarizes the distribution of metastatic sites across histologic ADCG, SCCG, and LCCG. For each site, counts and group percentages are shown (denominators are the n values in the headers). The Global *p*-value (3 × 2) comes from a two-sided Fisher–Freeman–Halton exact test comparing all three groups simultaneously. Pairwise *p*-values (ADCG vs. SCCG, SCCG vs. LCCG, ADCG vs. LCCG) are two-sided Fisher’s exact tests reported as raw and Holm-adjusted within row. Statistical significance should be judged on the adjusted *p*-values (α = 0.05).

Multinomial logistic regression revealed distinct organ-specific dissemination patterns according to histology (Table 3). Compared with squamous cell carcinoma, adenocarcinoma showed a markedly higher likelihood of presenting with brain metastases (RRR 3.74, 95% CI 1.48–9.45; *p* = 0.005, *p*_FDR_ = 0.053) and a moderate association with bone (RRR 2.27, 95% CI 1.07–4.80; *p* = 0.032, *p*_FDR_ = 0.159) and adrenal involvement (RRR 2.49, 95% CI 0.89–6.96). No significant associations were observed for liver spread. In contrast, pleural involvement was more often linked to squamous histology, with adenocarcinoma showing a lower relative risk (RRR 0.63, 95% CI 0.32–1.27). Large-cell carcinoma did not display consistent differences relative to squamous carcinoma across metastatic sites.

**Figure 1:**
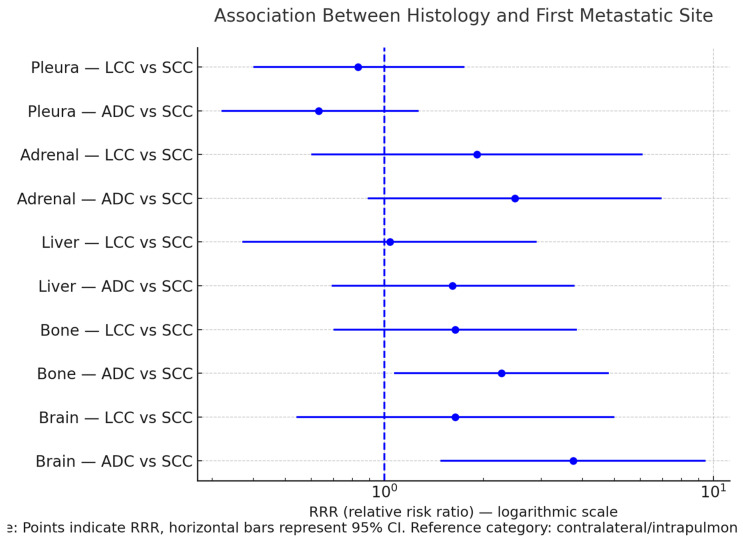
Association Between Histologic Subtype and First Metastatic Site at Diagnosis (Multinomial Model).

Figure 1 displays the unadjusted multinomial logistic model linking histologic subtype to the first metastatic site at diagnosis. Compared with SCC, ADC shows a clear enrichment for brain involvement and a tendency toward bone/adrenal, while pleural spread is relatively less likely; liver does not diverge meaningfully, and LCC shows no consistent deviation from SCC across sites. Taken together, Figure 1 supports histology-specific metastatic tropisms at baseline, but the patterns should be read cautiously given the unadjusted estimates and wider intervals for smaller subgroups (full estimates in Table 3).

Table 4 below consolidates, in a single view, the distribution of molecular alterations by histology alongside the organ-specific propensity of the first metastatic site at the time of adenocarcinoma (ADC) diagnosis. The aim is practical: histology-level prevalence contextualizes the cohort’s molecular profiling, while the distribution columns summarize early dissemination patterns by molecular status. While brain metastases predominated in EGFR-mutant tumors (55.6%), bone (22.2%), liver (11.1%), and pleural (11.1%) involvement were also observed. KRAS-mutant tumors most often metastasized to the liver (44.4%), followed by brain and bone (22.2% each) and adrenal glands (11.1%). ALK-rearranged tumors primarily involved bone (66.7%) and, less frequently, the brain (33.3%). Wild-type adenocarcinomas showed a heterogeneous pattern consistent with the overall histologic distribution. Findings should be interpreted cautiously where sample sizes are small, and “wild type” is defined strictly as EGFR/KRAS/ALK-negative. In this format, the table supports prioritizing baseline investigations according to histology and, for ADC, according to molecular status.

**Figure 2:**
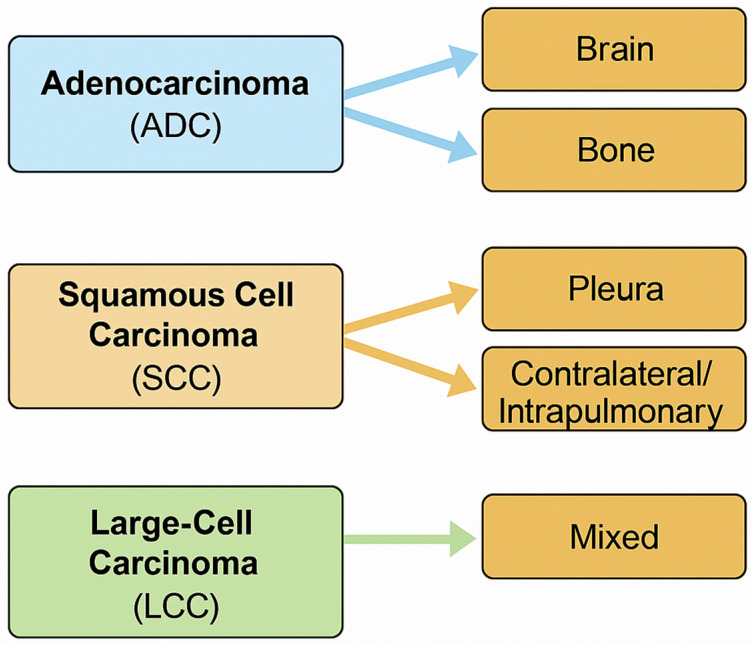
Graphical summary of histology-specific dissemination patterns at diagnosis in NSCLC.

To provide a concise visual overview of the observed dissemination patterns, Figure 2 summarizes the predominant first metastatic sites according to histologic subtype. ADC shows a marked tendency toward brain, bone, and adrenal involvement, whereas SCC more frequently spreads to the pleura or contralateral lung. LCC demonstrates a mixed distribution pattern, consistent with its heterogeneous biological behavior.

## 4. Discussion

Our study set out to answer a practical question with immediate implications for baseline staging: at the moment of a new NSCLC diagnosis, which organ is most likely to be the first metastatic site? We deliberately centered the analysis on the first site detected at diagnosis [5], rather than cumulative spread over time, and we stratified by histology [10]. Coupled with a uniform, high-intensity imaging protocol within a narrow pre-treatment window [28], this design keeps the signal we care about, what to look for first, close to the decisions clinicians must make in real clinics: how low to set the threshold for brain MRI [29], how carefully to scrutinize the adrenals, how aggressively to clarify contralateral/intrapulmonary disease, and how vigilant to be for pleural involvement. Because such choices are often shaped by habit and resource constraints, a disciplined readout tied to histology and routine tests can reduce both under- and over-staging.

What distinguishes this study from previous reports is its methodological consistency and focus on baseline dissemination patterns. Unlike earlier series that combined incident and subsequent metastases, we analyzed only the first metastatic site identified at diagnosis, using a standardized imaging protocol (contrast-enhanced CT, PET-CT, and brain MRI) applied uniformly within a ±7-day diagnostic window. This design minimized detection bias and provided a more accurate representation of initial metastatic routes. In addition, by integrating molecular data (EGFR, KRAS, ALK) with histology-specific patterns, this work delivers novel insights into the biological underpinnings of organotropism at presentation. Finally, we introduce the concept of a histology-informed triage strategy for imaging, which has direct implications for optimizing diagnostic efficiency and resource use in real-world oncology settings.

The demographic profile of our cohort broadly reflects that of the wider clinical population with advanced NSCLC seen across Europe. The median age being in the mid-sixties, the male predominance, and the distribution of histologic subtypes are consistent with data from large observational registries and SEER-based analyses. The higher proportion of active smokers within the squamous carcinoma group mirrors regional trends in Central and Eastern Europe, where tobacco exposure remains a major risk factor, particularly among men. Because our institution serves both urban and rural areas and functions as a referral center for multiple counties, the cohort captures a realistic cross-section of the patients encountered in routine oncology practice rather than a highly selected subgroup [2,4].

Institutional imaging protocols also had a clear influence on how metastatic patterns were detected and classified. At OncoHelp Medical Center, all patients underwent a standardized baseline work-up that included contrast-enhanced CT of the chest–abdomen–pelvis, whole-body FDG PET-CT, and brain MRI within seven days of histologic confirmation. This uniform, high-intensity imaging strategy likely improved detection of clinically silent metastases—particularly in the brain, bone, and adrenal glands—compared with cohorts where MRI or PET-CT are performed only when symptoms appear. Consequently, the slightly higher frequency of brain metastases among adenocarcinoma cases in our series probably reflects both genuine biological tendencies and better ascertainment through systematic imaging. While this approach minimizes under-staging bias, it also means that our results may differ from those reported in institutions where access to advanced imaging is less consistent [29].

What we found, in brief, is that histology matters for the first site at presentation. In our cohort, there was a global association between histology and the distribution of first metastatic sites [10,30]. Within that pattern, ADC showed a higher probability for brain [31] as the initial site in unadjusted multinomial models (a borderline signal after control for multiple testing), with directional tendencies toward bone [32] and adrenal glands [33]. SCC showed a more locoregional pattern, with pleural involvement more often emerging as the first site (retaining significance after multiplicity correction) and with contralateral/intrapulmonary disease more commonly encountered at baseline [5]. These directions of effect align well with prevailing clinical experience and mechanistic expectations, and they provide updated estimates derived from contemporary, systematic imaging that minimizes ascertainment gaps, especially for intracranial disease, osseous lesions, and adrenal metastases.

Placing our results in the context of what is already reported, the broad contours are consistent with the literature: ADC tends toward hematogenous spread early, involving the brain, bone, and adrenal glands [10,31,34], whereas SCC tends to remain locoregional longer, with pleura and contralateral/intrapulmonary involvement more prominent at presentation [5,35]. Where our data add nuance is in the magnitude of these signals and in the clarity afforded by focusing on the first site rather than pooling incident with prevalent metastases. Many historical cohorts mixed baseline and subsequent metastatic events or performed brain MRI selectively; as a result, organotropism at diagnosis can be blurred. By implementing a uniform baseline package (contrast-enhanced CT chest/abdomen/pelvis, whole-body PET-CT, and brain MRI within a tight window) and adjudicating a single first site using predefined rules, our estimates likely capture earlier, biologically proximate dissemination patterns. This also means that differences in magnitude versus older series are plausibly explained by more systematic brain imaging, better PET coverage, improved spatial resolution (with stage migration), and center-specific referral patterns [29,32,33,36].

From a biologic and morphopathologic standpoint, the observed tropisms are credible. ADC is frequently peripheral and grows in capillary-rich alveolar environments, where early access to the bloodstream can favor hematogenous dissemination [37]. Within a classical seed-and-soil framework, capillary-dense microenvironments such as the brain, bone marrow, and adrenal cortex supply stromal cues and niches that accommodate seeding and outgrowth [38]. Differences in adhesion programs (e.g., integrin repertoires), chemokine signaling axes, epithelial–mesenchymal transition, and immune editing can reasonably tilt colonization toward specific organs [39]. By contrast, SCC is often central, with direct continuity to major airways and rich lymphatic pathways; bulky endobronchial disease, segmental obstruction, and peribronchial spread make pleural involvement and contralateral/intrapulmonary disease at diagnosis easier to understand as early manifestations of local and lymphatic extension. We did not measure mechanistic biomarkers in this study; nonetheless, the convergence between what we observed clinically and what is expected biologically strengthens internal credibility.

The molecular context within ADC offered exploratory granularity that fits clinical intuition: EGFR-mutant tumors most often presented with the brain as the first site [40]; ALK-rearranged tumors clustered toward bone [41,42]; and KRAS-mutant tumors more frequently involved the liver first [43,44]. These signals, while aligned with prior clinical impressions, were based on small numbers and were not designed for confirmatory inference or full multiplicity control; moreover, in our dataset, positives for these three alterations occurred only within ADC, and the term ‘wild type’ was applied exclusively to cases tested and confirmed negative for EGFR, KRAS, and ALK. Cases lacking complete molecular testing were classified as ‘unknown biomarker status [43]. For interpretative consistency, only fully tested tumors were designated as “wild type”, whereas cases lacking complete molecular profiling were categorized as ‘unknown biomarker status, following standard oncology reporting conventions. The takeaway is therefore hypothesis-generating: it is plausible that genotype contributes meaningfully to organotropism at diagnosis in ADC, but formal confirmation requires larger, uniformly genotyped cohorts and prospective sampling frames.

These findings support the idea that histology alone cannot fully explain the biological heterogeneity underlying metastatic tropism in NSCLC. While the major dissemination patterns, such as the hematogenous spread typical of adenocarcinoma and the locoregional predominance of squamous carcinoma, reflect distinct tissue architectures, molecular alterations add an additional layer of precision. Integrating genotype-specific data such as EGFR, KRAS, and ALK status can refine risk estimates for organ-specific spread and guide more tailored imaging strategies. In this sense, histology provides a structural framework, but molecular profiling reveals the finer biological gradients that drive where metastases ultimately emerge. Future multicenter studies with larger, uniformly genotyped cohorts will be essential to validate and expand these molecular correlations.

The clinical translation is to adopt histology-informed triage within guideline pathways. For ADC, a lower threshold for brain MRI at diagnosis is reasonable, especially in symptomatic patients or when other risk indicators are present, paired with deliberate scrutiny of skeleton and adrenals [29,45]. When available, PET-CT remains an efficient whole-body survey; where it is not, a pragmatic combination of high-quality contrast-enhanced CT and targeted MRI for indeterminate adrenal lesions can implement similar priorities [46,47]. For SCC, the emphasis should shift toward rigorous clarification of contralateral/intrapulmonary disease and pleural involvement: meticulous cross-sectional imaging, tissue staging when appropriate (EBUS-TBNA or mediastinoscopy), and point-of-care ultrasound for effusions, reserving diagnostic thoracoscopy for selected cases [48,49]. We do not advocate replacing guideline algorithms; rather, we suggest triage within those algorithms, aligning initial testing intensity with the most likely biology of early spread suggested by histology.

The primary intent of a histology-informed diagnostic approach is to improve the efficiency, accuracy, and timeliness of baseline staging rather than to directly influence survival outcomes. By tailoring the intensity and focus of initial imaging to the most probable metastatic pattern, clinicians can reduce under-staging, minimize unnecessary investigations, and ensure that systemic treatment begins without delay. Although our findings do not imply that histology-guided imaging alone modifies prognosis in stage IV disease, a more precise baseline characterization may facilitate the early identification of patients with limited or oligometastatic involvement who could benefit from local ablative strategies, including surgery or stereotactic radiotherapy. In this way, optimized diagnostic pathways may indirectly contribute to better overall management and more personalized therapeutic decisions.

These results also inform diagnostic stewardship. Intensifying imaging indiscriminately at baseline can inflate incidental findings and trigger cascades of low-yield tests; conversely, under-staging can delay appropriate therapy [50]. A histology-anchored triage strategy offers a middle path: raise the intensity where yield is likely higher (e.g., brain MRI in ADC), temper it where yield is expected to be low, and tailor additional tests based on symptoms and simple clinical cues. This concept is scalable across resource settings. In constrained environments, anchoring on histology with judicious use of CT and targeted MRI can achieve a favorable balance between thoroughness and practicality, while in high-resource settings, a uniform PET-CT plus selective MRI strategy may be optimal [29].

A practical extension of this work is a simple triage heuristic for baseline staging. Histology serves as the anchor; symptoms and a handful of readily available cues modulate the threshold for specific tests. In ADC, neurologic symptoms or subtle cognitive changes could further lower the threshold for brain MRI [29]; bone pain or elevated alkaline phosphatase could nudge skeletal evaluation; and indeterminate adrenal nodules prompt targeted MRI characterization [51]. In SCC, persistent unilateral effusion, pleuritic pain, or extensive endobronchial disease should prompt early pleural clarification and thorough contralateral/intrapulmonary mapping [52]. Such a heuristic can be evaluated prospectively with process metrics (time to complete staging, proportion of low-yield tests) and clinical endpoints (change in management, time to treatment start, oncologic outcomes). Parallel cost-effectiveness analyses would clarify the budget impact of reallocating imaging intensity according to histology-informed risk [53].

A brief word on the LCC subset in our series. While included in all analyses and reported descriptively, the smaller sample constrains precision. Directionally, some patterns appear to overlap with ADC, but without the statistical weight to draw firm inferences. This underscores the value of pooled, multicenter datasets for less common histologies, where standardizing imaging and first-site adjudication could reveal reproducible organotropism at diagnosis.

The relatively small number of patients with large-cell carcinoma and with rare metastatic sites inevitably limits statistical power, leading to wide confidence intervals and loss of significance after multiple-testing adjustment. These results should therefore be interpreted as exploratory signals rather than confirmatory findings. We opted not to merge histologic categories or apply Bayesian shrinkage models in this initial analysis, as our primary goal was to preserve subtype-specific patterns with direct clinical interpretability. In future multicenter extensions of this work, larger sample sizes will enable more stable estimates and may justify hierarchical or Bayesian modeling to refine organ-specific probability distributions.

In sum, the first metastatic site at NSCLC diagnosis is not random. It can be anticipated using information already available at baseline, with histology as the organizing principle and routine tests as practical adjuncts. Aligning initial imaging with the expected pattern of early spread can increase diagnostic yield, shorten the path to treatment decisions, and promote responsible use of resources. Our estimates, derived from a standardized diagnostic window and explicit first-site assignment, provide a contemporary benchmark. The next step is prospective, multicenter validation that preserves these methodological strengths, examines the contribution of molecular and inflammatory markers in larger, uniformly profiled cohorts, and tests whether a histology-anchored triage strategy measurably improves both the efficiency and the outcomes of baseline staging.

### Strengths, Limitations and Future Directions

This study has several strengths that enhance both its credibility and its clinical relevance. The demographic structure of our cohort aligns closely with that of other real-world NSCLC populations, supporting the broader applicability of our findings. Minor regional differences—most notably the higher prevalence of smoking among men—are consistent with national epidemiologic patterns. The institutional imaging protocol, which required PET-CT and brain MRI at baseline for every patient, represents both a strength and a limitation: it reduces the risk of missed metastases but may also increase the apparent frequency of certain sites, particularly brain and bone, compared with studies that use more selective imaging. These aspects should be kept in mind when interpreting or extrapolating our results to centers with different diagnostic resources. The study focuses on the first metastatic site identified at baseline; a target directly aligned with decisions clinicians must make at diagnosis. By anchoring analyses in histology and augmenting them with routinely available clinical, imaging, and laboratory information, the work leverages variables that are accessible in most settings, improving the likelihood of adoption. The cohort is contemporary and real-world, reflecting current diagnostic pathways and practice patterns rather than historical protocols, and the inclusion criteria emphasize a clean baseline assessment before systemic therapy. The analytic approach prioritizes interpretability, reporting effect sizes with confidence intervals, while exploring interactions that could reveal clinically meaningful heterogeneity. Together, these features provide a coherent framework that can be incorporated into existing staging workflows without specialized infrastructure.

This study has several limitations. Its retrospective, single-center design introduces potential selection and information bias and may limit generalizability. Our strict inclusion criterion—mandatory CT chest–abdomen–pelvis, whole-body FDG PET-CT, and brain MRI within 7 days before treatment—reduces detection bias for intracranial and extrathoracic disease but likely selects fitter patients and, in edge cases, could misclassify the “first site” if progression occurred between scans. The operational rule used to adjudicate simultaneous findings, together with unavoidable inter-scanner and inter-reader variability, may also yield some misclassification, and very small or hypometabolic metastases can be missed even with modern imaging. Power was limited for certain subgroups (notably LCC and rare metastatic sites), leading to wide confidence intervals and attenuation after multiplicity correction. Estimates depend on the chosen reference outcome category (contralateral/intrapulmonary); using alternative references may shift emphasis. Finally, we lacked external validation and longitudinal endpoints, so findings should be viewed as baseline associations that merit confirmation in multicenter cohorts. Survival outcomes were not analyzed in the present study because follow-up data were incomplete at the time of cutoff. The focus was deliberately limited to baseline diagnostic patterns to maintain internal consistency and avoid treatment-related confounding. Nevertheless, we plan to extend the current work once longitudinal data mature, to examine whether the histology-specific patterns of early dissemination observed here also translate into meaningful differences in progression-free and overall survival.

Future work should pursue prospective, multicenter validation with standardized imaging protocols, including a uniform strategy for brain MRI at diagnosis, to reduce ascertainment bias and permit robust external calibration. A practical next step is the development of a parsimonious risk tool that combines histology with a limited set of incremental predictors—such as select genomic alterations and CBC-derived inflammatory indices—optimized for discrimination and calibration and evaluated via decision-curve analysis to quantify net clinical benefit across plausible threshold probabilities. Cost-effectiveness analyses are warranted to determine whether risk-adapted imaging strategies can reduce unnecessary tests while maintaining or improving detection of actionable disease. Implementation studies embedded in routine care, ideally with EHR integration and simple clinical calculators, should assess process metrics (time to complete staging, number of tests) and patient-centered outcomes (time to treatment, unplanned admissions). Methodologically, future models would benefit from pre-registration, transparent handling of missingness, and fairness assessments across age, sex, and comorbidity strata. Finally, exploratory integration with radiomics and ctDNA could refine risk estimates at the margins, while mechanistic correlative studies—probing adhesion, chemokine signaling, and microenvironmental features—may help explain organotropism and identify targets for intervention.

## 5. Conclusions

In our stage IV NSCLC cohort (n = 364; 2020–2024), histology was associated with the distribution of the first metastatic site at diagnosis (3 × 6 global test *p* = 0.013). In unadjusted multinomial models, adenocarcinoma vs. squamous cell carcinoma showed a higher probability for brain as first site (FDR-adjusted *p* = 0.053), while pleura as a first site was more frequent in squamous carcinoma on post hoc testing (adjusted *p* = 0.008). Within adenocarcinoma, exploratory molecular subgroups showed: EGFR 10/18 (55.6%) brain; KRAS 4/9 (44.4%) liver; ALK 3/3 (100%) bone.

## Figures and Tables

**Table 1:** Baseline characteristics of patients with NSCLC by histologic subtype.

Characteristic	ADCG (N = 164)	SCCG (N = 112)	LCCG (N = 88)	*p* Value	*p* Value ADCG vs. SCCG	*p* Value SCCG vs. LCCG	*p* Value ADCG vs. LCCG
Mean age (years, ±SD)	64.1 ± 9.3	66.8 ± 8.1	65.4 ± 8.7	0.045	0.011	0.360	0.405
Male sex, n (%)	101 (61.6%)	78 (69.6%)	58 (65.9%)	0.383	0.199	0.703	0.726
Active smokers, n (%)	92 (56.1%)	92 (82.1%)	56 (63.6%)	<0.001	<0.001	0.019	0.395
BMI, mean (kg/m^2^, ±SD)	25.3 ± 3.8	26.1 ± 3.7	25.9 ± 4.1	0.210	0.083	0.768	0.361
ECOG performance status 0–1, n (%)	110 (67.1%)	65 (58.0%)	54 (61.4%)	0.300	0.129	0.721	0.479
Hypertension, n (%)	64 (39.0%)	47 (42.0%)	34 (38.6%)	0.869	0.707	0.729	>0.999
Diabetes mellitus, n (%)	40 (24.4%)	25 (22.3%)	22 (25.0%)	0.201	0.773	0.833	>0.999

Continuous variables are reported as mean ± SD (or median [IQR] if distributions are skewed); categorical variables as n (%). Global comparisons across the three groups used one-way ANOVA/Kruskal–Wallis (continuous) and χ^2^/Fisher’s exact tests (categorical). Percentages use the denominators indicated in the column headers.

**Table 2:** Distribution of metastatic sites according to histopathological subtype of NSCLC.

Metastatic Site	Adenocarcinoma (n = 164)	Squamous Cell Carcinoma (n = 112)	Large-Cell Carcinoma (n = 88)	Global *p*-Value	*p* Value ADCG vs. SCCG (Raw)/(Adjusted)	*p* Value SCCG vs. LCCG (Raw)/(Adjusted)	*p* Value ADCG vs. LCCG Raw)/(Adjusted)
Brain	28 (17.1%)	7 (6.2%)	8 (9.1%)	0.017	0.009/0.028	0.590/>0.999	0.092/0.276
Bone	34 (20.7%)	14 (12.5%)	16 (18.2%)	0.214	0.105/0.315	0.320/0.959	0.741/>0.999
Liver	19 (11.6%)	11 (9.8%)	8 (9.1%)	0.839	0.698/>0.999	>0.999/>0.999	0.671/>0.999
Adrenal glands	16 (9.7%)	6 (5.3%)	8 (9.1%)	0.404	0.258/0.774	0.404/>0.999	>0.999/>0.999
Contralateral lung/intrapulmonary	46 (28.1%)	43 (38.4%)	30 (34.1%)	0.185	0.088/0.264	0.557/>0.999	0.319/0.956
Pleura	21 (12.8%)	31 (27.7%)	18 (20.4%)	0.008	0.003/0.008	0.252/0.755	0.143/0.429

**Table 3:** Multinomial logistic regression (UNADJUSTED) for the association between histology (ref.: SCC) and the first metastatic site (ref. outcome: Contralateral/Intrapulmonary).

Site (vs. Ref.: Contralateral/ Intrapulmonary)	Comparison (Histology)	RRR (95% CI)	*p*	pFDR
Brain	ADC vs. SCC	3.74 (1.48–9.45)	0.005	0.053
	LCC vs. SCC	1.64 (0.54–5.00)	0.386	0.483
Bone	ADC vs. SCC	2.27 (1.07–4.80)	0.032	0.159
	LCC vs. SCC	1.64 (0.70–3.85)	0.258	0.389
Liver	ADC vs. SCC	1.61 (0.69–3.78)	0.270	0.389
	LCC vs. SCC	1.04 (0.37–2.90)	0.937	0.937
Adrenal	ADC vs. SCC	2.49 (0.89–6.96)	0.081	0.270
	LCC vs. SCC	1.91 (0.60–6.08)	0.272	0.389
Pleura	ADC vs. SCC	0.63 (0.32–1.27)	0.196	0.389
	LCC vs. SCC	0.83 (0.40–1.75)	0.629	0.699

RRR (95% CI), *p*, and *p*_FDR_ are reported. Multiple testing was controlled with Benjamini–Hochberg across the 10 component comparisons (5 sites × 2 histology contrasts); pairwise significance is judged on *p*_FDR_ (α = 0.05). The global test for the histology–first site association (3 × 6 G-test) was *p* = 0.013. Estimates are derived from the distributions in Table 2; model unadjusted for covariates. Abbreviations: RRR—relative risk ratio; CI—confidence interval; FDR—false discovery rate; SCC—squamous cell carcinoma; ADC—adenocarcinoma; LCC—large-cell carcinoma.

**Table 4:** Molecular alterations and predominant first metastatic site at diagnosis.

Molecular Alteration	ADC (n = 164) n (%)	SCC (n = 112) n (%)	LCC (n = 88) n (%)	Total (n = 364) n (%)	Distribution of First Metastatic Sites at Diagnosis (ADC Only)	n/N (%)	95% CI (Wilson)
EGFR mutation	18 (11.0%)	0 (0.0%)	0 (0.0%)	18 (4.9%)	Brain 10 (55.6%), Bone 4 (22.2%), Liver 2 (11.1%), Pleura 2 (11.1%)	10/18 (55.6%)	33.7–75.4%
KRAS mutation	9 (5.5%)	0 (0.0%)	0 (0.0%)	9 (2.5%)	Liver 4 (44.4%), Brain 2 (22.2%), Bone 2 (22.2%), Adrenal 1 (11.1%)	4/9 (44.4%)	18.9–73.3%
ALK rearrangement	3 (1.8%)	0 (0.0%)	0 (0.0%)	3 (0.8%)	Bone 2 (66.7%), Brain 1 (33.3%)	3/3 (100.0%)	43.8–100.0%
EGFR/KRAS/ALK-negative (tested)	134 (81.7%)	112 (100.0%)	88 (100.0%)	334 (91.8%)	Mixed: Pleura 30 (22.4%), Contralateral/Intrapulmonary 40 (29.9%), Bone 25 (18.7%), Brain 22 (16.4%), Liver 17 (12.7%)	—	—

Prevalence values are reported as n (%) using histology-specific denominators (ADC, SCC, LCC) and overall, for the total cohort. The columns “Top first site at diagnosis (ADC only)”, “n/N (%)”, and “95% CI (Wilson)” refer exclusively to the ADC subgroups. “Wild type” indicates negative for EGFR, KRAS, and ALK; other alterations are not included. Estimates for “Top first site” are exploratory and unadjusted for multiple comparisons. Abbreviations: ADC—adenocarcinoma; SCC—squamous cell carcinoma; LCC—large-cell carcinoma; CI—confidence interval. Molecular testing for EGFR, KRAS, and ALK was performed in all or nearly all patients with adenocarcinoma at baseline, in line with institutional standards. Percentages for molecular subgroups reflect the proportion of tested adenocarcinomas, not the total NSCLC cohort. Only tumors tested for all three biomarkers (EGFR, KRAS, and ALK) and confirmed negative were designated as “wild type.” Cases not tested for one or more biomarkers were classified as “unknown biomarker status”.

## Data Availability

The data presented in this study are available upon request from the corresponding author. The data are not publicly available due to hospital policy.
